# Implementation of Food is Medicine Programs in Healthcare Settings: A Narrative Review

**DOI:** 10.1007/s11606-024-08768-w

**Published:** 2024-04-25

**Authors:** Bailey Houghtaling, Eliza Short, Carmen Byker Shanks, Sarah A. Stotz, Amy Yaroch, Hilary Seligman, James P. Marriott, Jenna Eastman, Christopher R. Long

**Affiliations:** 1 Gretchen Swanson Center for Nutrition, Omaha, NE USA; 2 Department of Human Nutrition, Foods, and Exercise, Virginia Tech, Blacksburg, VA USA; 3 Department of Food Science and Human Nutrition, Colorado State University, Fort Collins, CO USA; 4 Division of General Internal Medicine and Center for Vulnerable Populations, University of California San Francisco, San Francisco, CA USA

**Keywords:** food is medicine, implementation science, implementation strategy, healthcare.

## Abstract

Food is Medicine (FIM) programs to improve the accessibility of fruits and vegetables (FVs) or other healthy foods among patients with low income and diet-related chronic diseases are promising to improve food and nutrition security in the United States (US). However, FIM programs are relatively new and implementation guidance for healthcare settings using an implementation science lens is lacking. We used a narrative review to describe the evidence base on barriers and facilitators to FIM program integration in US healthcare settings following the Exploration, Preparation, Implementation, and Sustainment (EPIS) Framework. Evidence surrounding the EPIS *Inner Context* was a focus, including constructs *Leadership*, *Organizational Characteristics*, *Quality and Fidelity Monitoring and Support*, *Organizational Staffing Processes*, and *Individual Characteristics*. Peer-reviewed and grey literature about barriers and facilitators to FIM programs were of interest, defined as programs that screen and refer eligible patients with diet-related chronic disease experiencing food insecurity to healthy, unprepared foods. Thirty-one sources were included in the narrative review, including 22 peer-reviewed articles, four reports, four toolkits, and one thesis. Twenty-eight sources (90%) described EPIS *Inner Context* facilitators and 26 sources (84%) described FIM program barriers. The most common barriers and facilitators to FIM programs were regarding *Quality and Fidelity Monitoring and Support* (e.g., use of electronic medical records for tracking and evaluation, strategies to support implementation) and *Organizational Staffing Processes* (e.g., clear delineation of staff roles and capacity); although, barriers and facilitators to FIM programs were identified among all EPIS *Inner Context* constructs. We synthesized barriers and facilitators to create an EPIS-informed implementation checklist for healthcare settings for use among healthcare organizations/providers, partner organizations, and technical assistance personnel. We discuss future directions to align FIM efforts with implementation science terminology and theories, models, and frameworks to improve the implementation evidence base and support FIM researchers and practitioners.

**Supplementary Information:**

The online version contains supplementary material available at 10.1007/s11606-024-08768-w.

## BACKGROUND

While higher fruit and vegetable (FV) intake is associated with positive health outcomes, ^1^ most adults in the United States (US) do not meet FV intake recommendations as advised by the Dietary Guidelines for Americans. ^2,3^ Racial and ethnic minority populations with low income face greater challenges in accessing FVs due to structural inequities, ^4–8^ contributing to a disproportionate burden of diet-related chronic disease. ^1,9^ Food and nutrition security strategies that ensure all Americans have consistent access to healthy foods to prevent or treat diet-related chronic disease^10^ are gaining attention as a national priority^11^ to supplement other forms of federal food assistance, such as the Supplemental Nutrition Assistance Program (SNAP). ^12^

Food is Medicine (FIM) programs are one such approach, typically calling for healthcare providers such as physicians and allied health professionals to screen patients with diet-related chronic disease for food insecurity and refer to reduced cost or free nutritious food, especially FVs, often for redemption at an off-site location like food retail sites or food banks/pantries. ^11,13–15^ FIM programs are promising, with some estimating that national implementation of FV prescriptions could save nearly 40 billion dollars in healthcare costs and prevent over 250,000 cardiovascular disease instances. ^16^ As such, funders are increasing investments in FIM to expand reach and elucidate program impacts. The US Department of Agriculture is one example that funds FV prescriptions nationwide through the Gus Schumacher Nutrition Incentive Program (GusNIP) and supports the National Training, Technical Assistance, Evaluation, and Information Center (NTAE) to provide GusNIP grantees with implementation and evaluation support. ^17^

However, many healthcare organizations, especially those that serve patients with low incomes, face challenges in the funding, staff time, technology, and experience or knowledge necessary for deploying FIM programs. ^14,18–21^ Because these programs are relatively new and the literature base is rapidly growing, it is critical to understand gaps and opportunities to FIM adoption, implementation, and sustainability (herein “integration”) in diverse US healthcare contexts (e.g., rural, tribal), ^22–26^ especially amid calls for scaling. ^16,23,27,28^ Implementation science is the study of evidence-based innovation integration into real-world settings to ensure benefits are realized, barriers overcome, and assets built upon. ^23,29^ Importantly, implementation science research demonstrates contextual factors such as the characteristics of organizations, staff, leadership, and processes are often predictive of efforts to integrate evidence-based innovations in healthcare settings. ^23–26^

We used a narrative review to describe the state of evidence on barriers and facilitators to FIM program integration in healthcare settings following the Exploration, Preparation, Implementation, and Sustainment (EPIS) Framework. ^24,25^ Results were used to (1) create an EPIS-informed checklist for FIM programs in healthcare settings for use by providers and partner organizations and (2) to discuss gaps and opportunities for research, practice, and policy.

## METHODS

Experts in public health nutrition, implementation science, and medicine conducted a narrative literature review to examine barriers and facilitators to FIM program integration in healthcare settings. Narrative reviews are useful amid a newer literature base and when expert synthesis could favorably impact the quality and reporting of future research and practice approaches. ^30,31^ Co-authors drew on versatile expertise to offer research, practice, and policy guidance for FIM programs in healthcare settings.

### Narrative Review Scope and Source Identification

English-language sources, including peer-reviewed and grey literature that focused on barriers and facilitators to FIM programs in US healthcare settings, regarding the screening and referral of eligible patients with diet-related chronic disease experiencing food insecurity to healthy (e.g., FVs or other healthy food), unprepared foods, ^13^ were included as narrative review evidence. Prepared food interventions, such as medically tailored meals, were not included. ^13^

Sources were identified using several mechanisms. We reviewed included sources among three NTAE-authored systematic scoping reviews that were designed to capture the breadth of nutrition incentive and produce/healthy food prescription literature, including the evaluation of produce/healthy food prescription programs, ^32^ the evaluation of nutrition incentive programs, ^33^ and partner perspectives regarding these programs. Together, these reviews used key terms that were structured and tested by research librarians across eight databases (PubMed, Ovid Medline, CINAHL, PsychInfo, Web of Science, Agricola, CAB Direct, and ProQuest Global), resulting in over 8,000 total records to date. Grey literature sources were also reviewed per the search strategies. Additional details about these systematic searches can be found in publicly posted protocols^32,33^ and forthcoming publications. Additionally, for the narrative review, these prior searches were supplemented by reviewing resources developed for FIM practitioners on the Nutrition Incentive Hub webpage, ^34^ as well as emerging literature on this topic identified by monitoring listservs and publication alerts.

### Framework

Evidence was synthesized using the EPIS Framework, a process and determinant framework that is widely used in implementation science^24,25,35–37^ and recently recommended for the FIM movement. ^28^ The four EPIS process phases are *Exploration* (e.g., identifying FIM program need and type), *Preparation* (e.g., understanding FIM program barriers and facilitators to inform implementation strategies^22,23^), *Implementation* (e.g., implementation of the FIM program and implementation strategies^22,23^), and *Sustainment* (e.g., continuation over time). ^25^ Implementation strategies are interventions aimed at improving innovation integration among implementors and implementing organizations, for example, and include at least 73 strategies that have been proposed for healthcare settings. ^22^ EPIS categorizes barriers and facilitators across the four phases, from *Exploration* to *Sustainment*, using four domains–*Outer Context*, *Bridging Factors*, *Innovation*, and *Inner Context*. ^24,25^ We focus on barriers and facilitators specific to EPIS *Inner Context* constructs, including *Leadership*, *Organizational Characteristics*, *Quality and Fidelity Monitoring and Support*, *Organizational Staffing Processes*, and *Individual Characteristics* (Table 1), to offer rapid and pragmatic guidance for FIM program integration in healthcare settings.

**Table 1:** Exploration, Preparation, Implementation, Sustainment (EPIS) Framework Definitions^*^ Pertaining to the EPIS Inner Context and Food is Medicine (FIM)^†^ Programs

Leadership	Characteristics or behaviors of leaders within a healthcare organization or setting with oversight or decision-making responsibilities that may influence FIM program adoption, implementation, or sustainment
Organizational Characteristics	Characteristics of the healthcare organization or setting such as the organizational mission and climate, inter-organizational networks, and existing structures or processes that may influence FIM program adoption, implementation, or sustainment
Quality and Fidelity Monitoring and Support	Processes or procedures used to ensure or monitor active program delivery that may influence FIM program adoption, implementation, or sustainment
Organizational Staffing Processes	Processes or procedures regarding hiring, retention, and training of healthcare providers or staff that may influence FIM program adoption, implementation, or sustainment
Individual Characteristics	Shared or unique characteristics or attitudes, beliefs, and perceptions of healthcare providers or staff that may influence FIM program adoption, implementation, or sustainment

^*^Based on definitions presented by Moullin et al., 2019^25^ and modified for the healthcare context

^†^Refers to FIM programs based in a U.S. healthcare context that screen and refer patients to healthy, unprepared foods

### Data Charting and Interpretation

Information extracted from each source included a description of the source type, study design, program location, partner organizations, and the FIM program. Additionally, results on barriers and facilitators, based on source framing, were extracted to EPIS *Inner Context* constructs by best fit. Two authors were involved in data extraction and categorization agreement (JM, BH); decision-making was iterative during the table-making and interpretation phases (JM, BH, ES). Results are presented in narrative form with accompanying supplemental data tables. An EPIS-informed implementation checklist (Text Box 1) synthesizes the barriers and facilitators to FIM program integration in healthcare settings and was created to support healthcare organizations/providers, partner organizations, and technical assistance personnel who are interested in or actively integrating FIM programs in healthcare settings.

**Text Box 1**. Food is Medicine (FIM) implementation checklist for healthcare settings

**Table:** 

**Leadership**
• Ensure buy-in, enthusiasm, and commitment for FIM programs from healthcare leadership and program implementation leaders from the earliest stages and onward • Identify and prepare program/clinical “champions” at the organizational and program levels to manage project-level responsibilities and coordinate higher systems-level strategies • Obtain letters of support from healthcare administration to overcome data sharing, electronic medical record (EMR), and Institutional Review Board (IRB) barriers • Recruit, designate, and train healthcare providers that are knowledgeable of diverse clinic roles to become implementation leaders **Organizational Characteristics** • Allow adequate time prior to FIM program implementation to plan organizational logistics (e.g., staffing, cost, advertising/outreach, physical space, safe food storage, alignment with existing workflow) and obtain buy-in from clinic departments • Conduct education and outreach visits with clear dialogue about FIM programs aligned with organizational missions • Establish frequent communication methods between implementing organizations (e.g., food bank and healthcare partners) to facilitate programming, provide updates, create clear expectations, and allow for interactive problem solving • Identify organizational and community partners that are in alignment with FIM program goals, including nontraditional program sites as potential implementing organizations (e.g., school-based health center) • Obtain data sharing, partnership, and reporting agreements that align with patient privacy laws (e.g., memorandum of understanding) between implementing partner organizations • Proactively address institutional biases (using institutional policies and practices, advocacy priorities, and training) to ensure equitable and anti-racist implementation of FIM programs **Quality and Fidelity Monitoring and Support** • Build closed-loop communication and data sharing systems for agreed upon FIM program and patient outcomes of interest between implementing organizations • Build patient awareness using educational materials to encourage demand for and use of the FIM program • Identify a consistent screening tool/process to identify eligible patients and set-up an existing EMR to allow for seamless screening, referral, tracking, coding, and billing related to the FIM program • Implement regular communication streams (e.g., monthly conference calls, checklists to guide patient-provider interactions), clinician reminders (e.g., memos, EMR notes), and capture and share implementation feedback about referral rates and performance during the implementation period to facilitate FIM programs and allow for troubleshooting. Partnering with a research or technical assistance team may be beneficial • Start new FIM program initiatives or components (e.g., health outcome data collection) with one clinic prior to spreading to new sites • Utilize quality improvement tools such as RACI (Responsible, Accountable, Consulted, and Informed) or develop and use other FIM program guides with clear instructions to ensure uniform implementation across all healthcare partners **Organizational Staffing** • Ensure healthcare partner compensation for their time investment • Identify or hire champions (e.g., registered dietitian nutritionists (RDNs), nursing or healthcare technicians/assistants, social workers/case managers) to lead and coordinate FIM programs in alignment with standard healthcare procedures • Include all healthcare providers and staff (e.g., physicians, nurses, RDNs, administrators, social workers) in planning for a successful FIM program • Offer healthcare providers and staff ongoing training and support materials, such as an implementation guide and periodic training that focuses on: program roles and responsibilities; screening tools/eligibility; referral process; program benefits; required documentation; FIM program logistics; and nutrition training • Plan for time and capacity constraints among healthcare providers involved in the FIM program. Hiring outside support or identifying volunteers (e.g., allied health professionals, nursing students) may be needed • Promote the flexibility of FIM programs to accommodate patient and provider needs (e.g., helping patients with no internet access complete a survey, providing group patient education, hiring multilingual staff members) • Shift roles and responsibilities within clinical teams to facilitate FIM programs is recommended. For example, hiring or designating one staff member (e.g., RDN) to facilitate FIM program implementation can help to improve fidelity and patient engagement and designating allied health professionals (e.g., certified nursing assistants, medical assistants, community health workers) instead of physicians to conduct screening and enrollment may also be beneficial **Implementor Characteristics** • Locate program champions with strong communication skills and enthusiasm • Sharing favorable perceptions of FIM programs may help to ensure implementor buy-in. For example, physicians and other allied health and support professionals have expressed program and job satisfaction, improved patient-provider relationships, and greater awareness and agency to address patient food insecurity because of implementing FIM programs

## RESULTS

Thirty-one sources published between 2014 and 2024 were included in the narrative review (Supplemental Table 1), ^18,38–67^ with 84% being published since 2019. ^18,38–41,43,45,47–56,58–60,62–67^ Sources included 22 peer-reviewed articles (71%),^18,38,39,41,43,44,46,48–53,55,56,58,60,61,63,64,66,67^ four reports, ^45,47,59,62^ four toolkits, ^40,42,54,65^ and one thesis. ^57^ Partners for FIM programs in healthcare settings (when applicable or described) included the following: local food system and retail partners; ^40,42,44–46,48,50,53,57,58,60^ food banks and food pantries; ^38,43,47,51,52,55,56,59,66,67^ community-based or national organizations; ^40,42,50,53,57,60,64,65^ health departments; ^38,49,50,60,61^ community residents/experts; ^40,46,48,60^ academia; ^46,49,61^ land-grant university-based Extension; ^49,61^ early childcare education; ^53^ social services; ^45^ and a technical assistance provider. ^53^

Twenty-eight sources (90%) described EPIS *Inner Context* facilitators (Supplemental Table 2), and 26 sources (84%) described barriers to FIM programs (Supplemental Table 3). Facilitator and barrier findings related to FIM program integration in healthcare settings are separately described below and are combined for ease of use in the EPIS-informed implementation checklist (Text Box 1).

### Implementation Facilitators

#### Leadership

Fourteen sources described healthcare or implementation leadership (Table 1) as facilitators to FIM programs. ^18,40,42,43,47,49,53,54,56,59,60,62,65,66^ Leveraging champions for FIM programs among multiple levels of leadership^40,43,54,59,65,66^ and between partner organizations^56^ was key. Early engagement and efforts to ensure healthcare leadership buy-in^42,47,54,60,62,65^ or institutional and department approval^40,53^ were important. Framing the need for FIM programs to resonate with leadership values was described as necessary for buy-in^42,49,56^ and to prevent future monitoring and evaluation barriers. ^18^ One source noted that program leaders should have in-depth institutional knowledge of organizational roles/procedures. ^49^

#### Organizational Characteristics

Twelve sources described healthcare organizational characteristics (Table 1) as facilitators. ^38,40,42,47,51,53–56,59,63,66^ Organizational mission or vision should be woven into the foundation of FIM programs to determine compatibility and readiness between and within implementing organizations. ^40,53,56,63^ Additional facilitators included the clinic location, such as clinic proximity to priority populations and food redemption sites, the alignment with organizational missions to deliver culturally-relevant programs, and having the infrastructure to leverage institutional resources or use telemedicine to reach remote communities. ^42,53,66^ Inter- and intra-organizational communication was helpful in coordinating patient care, building trust, and strengthening organizational relationships. ^38,47,51,59^ Further, healthcare providers at times reached out to food banks to establish new partnerships or to replicate existing FIM programs, ^47,55^ which may evidence program alignment with organizational missions. One source recommended exploring healthcare partnerships with non-traditional sites (e.g., school-based health clinics) in rural areas and creating data sharing and partnership agreements between organizations. ^54^ Last, framing FIM as part of a value-based healthcare strategy and strengthening ties with other community programs was described as important to address food and nutrition insecurity. ^38,51^

#### Quality and Fidelity Monitoring and Support

Twenty-one sources described facilitators for the active delivery of FIM programs in healthcare settings (Table 1). ^18,38,39,42–44,46–49,51,53,54,56,57,59–61,63,65,66^ The use of electronic medical records (EMRs) was a key facilitator to efficiently screen and identify eligible patients, ^39,43,46,60,65,66^ track program fidelity and health outcomes, ^39,49^ and for program integration between healthcare organizations. ^53^ Two sources suggested electronic and online scheduling tools to aid patient enrollment. ^42,48^ Other methods to promote FIM program adoption, referral, and engagement included piloting programs prior to full-scale implementation, ^63^ champions encouraging referrals during patient visits, ^43,65^ universal patient screening for equitable program access, ^56^ validated screeners in medical intake forms, ^18^ diverse patient marketing techniques like texts or clinic marketing, ^18^ and the recommendation that providers emphasize FIM as equally important to pharmaceutical medications for diet-related chronic disease. ^57^ Some facilitators of data sharing across organizations included assigning staff to manage data reporting^59^ and working together to establish outcomes of interest and data sharing procedures^47,54^; however, obtaining EMR data may be easier when implementing FIM within one healthcare system due to privacy laws. ^65^

Several communication strategies facilitated FIM programs, including reminders, support, and problem solving, using teleconferences between healthcare implementors and partners, ^38,44,46,49,57,61^ and email updates with program referral, enrollment, and redemption data to encourage program use. ^42,44,46,57^ Additionally, facilitators included establishing a team charter agreement for implementation sites, ^53^ quality improvement tools for uniform FIM program implementation, ^42^ and providing clinician metrics regarding performance in screening and addressing patients’ needs. ^51^ Last, hiring full-time staff to coordinate all aspects of FIM programs was a facilitator to overcome patient engagement challenges and contribute to higher fidelity. ^18,57^

#### Organizational Staffing Processes

Twenty-three sources noted healthcare staffing as facilitators to FIM programs (Table 1). ^18,38–43,46,47,49–51,53,54,56–58,60–62,64–66^ The importance of team collaboration with the entire healthcare staff to build trust and open communication was noted, ^38,53,60^ in addition to identifying a champion lead^40^ and clearly defining staff roles for FIM programs. ^42,57,60,66^ Many allied healthcare professionals held key roles in streamlining FIM program screening, enrollment, and implementation, including medical assistants, certified nursing assistants, registered dietitian nutritionists (RDNs), dietetic technicians, social workers, and community health workers. ^18,40–43,46,53,54,64^ Having bilingual staff distribute prescriptions also helped improve program reach. ^47,50^ Further, training for FIM program delivery staff improved aspects of implementation in several ways, including improving staff knowledge about team roles, ^18,41,49,60^ facilitating the connection between food insecurity and patient health, ^41,56,62,65^ improving confidence to discuss FIM programs with patients, ^40,42,57^ and increasing the number of patient screenings and enrollments. ^47^ Some sources discussed the importance of maintaining appropriate number of staff during program growth, including recruiting students or volunteers. ^47,51,57,66^ Also discussed were facilitators to align FIM programs with internal workflows, ^49,51,61^ including combining enrollment with a regular nutrition visit to improve clinician productivity, ^39^ delivering group education to overcome workload challenges, ^58^ and providing flexible programming to meet needs. ^47^ Last, healthcare providers’ devotion of time and resources for FIM partnerships was a facilitator, ^56^ which can be supported through compensation for time and effort. ^47^

#### Individual Characteristics

Thirteen sources described individual characteristics of providers in healthcare organizations (Table 1) as FIM program facilitators. ^18,38–41,44,46,48,49,58,59,61,64^ Key facilitators included providers’ excitement about offering FIM programs and the perception that program engagement enhanced job satisfaction and improved self-efficacy for addressing patient food insecurity. ^18,38,46,48,58,61^ Providers’ enthusiasm also helped to improve program sustainability and cultural appropriateness. ^49^ Champions with strong communication skills^40^ and allied healthcare staff (e.g., care managers, CHWs, and RDNs) with positive perceptions of FIM programs ^39^ were also facilitators. Multiple sources highlighted that providers perceived FIM programs to positively impact patients, including the management of diet-related chronic disease and patient care, ^18,44,48,59,64^ and that FIM programs had a relative advantage over other resources for patient food insecurity. ^48,61^ Engagement in FIM programs helped providers feel more comfortable prescribing FVs, discussing and addressing barriers to healthy eating, and was perceived to improve provider-patient relationships. ^18,41,46,61^

### Implementation Barriers

#### Leadership

Three sources reported leadership barriers (Table 1) for FIM programs related to obtaining leadership buy-in and support for FIM programs at all organizational levels. ^39,59,60^

#### Organizational Characteristics

Seven sources reported healthcare organizational characteristics (Table 1) as barriers to FIM programs. ^40,45,52,55,56,59,66^ Common barriers included a lack of adequate infrastructure for FIM programs, such as insufficient space to store fresh FVs, the misalignment of FIM programs with existing care procedures, and under-allocation of organizational resources like the funding, time, or staff needed to build and maintain an onsite food pantry. ^40,52,55,56,59,66^ Equitable access to FIM programs amid healthcare system biases (e.g., systemic racism) was also a noted challenge, with recommendations for training and institutional policies to overcome this barrier. ^45^ Providers’ perceptions of administrative burden also hindered FIM partnerships and programs. ^55^ Furthermore, priority alignment and communication between implementation and partner organizations was a noted barrier, including limited time and resource investments for FIM programs in smaller healthcare settings and misaligned perspectives about the types of food offered to FIM program participants. ^56,59^

#### Quality and Fidelity Monitoring and Support

Seventeen sources reported barriers to ensuring or monitoring the active delivery of FIM programs in healthcare settings (Table 1). ^18,39–41,43,45,47–50,52,55,56,59,62,63,65^ Data sharing challenges between co-implementing organizations, such as food banks and healthcare sites, to assess program impacts were notable, including concerns about patient privacy laws and/or sharing data with government entities, staff time and capacity to report data, and conflicting data sharing procedures between healthcare systems implementing FIM programs. ^18,40,43,45,47,56,59,63,65^ Other barriers related to patient food security screening and referral, including a lack of standardized screening tools and integration into EMRs, lower priority for screening and referrals resulting in poor patient understanding of the FIM program, and a lack of a formal or digital referral process between implementing partners. ^40,41,43,47,55,59^ EMRs were described as key to improving FIM program documentation through coding, billing, and ultimately expanding reimbursement. However, there were reported challenges in needing to use multiple EMR platforms to view FIM program data versus other EMR outcomes, and using EMRs to track or evaluate FIM programs was described as time-intensive and costly. ^39,40,45,49,50,65^ With EMR access available only within a clinic, one source reported challenges in relying on a paper-based tracking system when distributing prescriptions in community settings. ^50^ Other FIM program delivery challenges included a desire for close-loop referrals, a lack of quality improvement metrics associated with program implementation, communication challenges during implementation that reduced provider enthusiasm to enroll patients, and a need for a clear communication strategy to ensure consistent dietary messages between RDNs and healthcare providers. ^43,48,52,65^ Last, ensuring patient engagement with a FIM program was a noted challenge due to program requirements to attend nutrition classes and health check-ins (beyond screening) to receive FVs. ^62^

#### Organizational Staffing Processes

Twenty sources reported organizational staffing barriers to FIM programs (Table 1), ^18,39–41,43,44,46–50,52,55,58–60,62–65^ mainly regarding limited staff time and capacity for FIM programs, ^18,39,40,43,44,47,49,50,52,58,59,63–65^ especially for programs relying on hand-written prescriptions. ^50^ A system to incorporate additional staff for screening, enrollment, and implementation was needed. ^39,40,43^ However, added staff time was described to increase implementation costs^39^, and staff involvement in the process of distributing food or referring to food assistance resources could be time-consuming and a distraction to regular patient care. ^47,52,59^ Seven sources described barriers related to a lack of training, ^18,41,46–48,60,62^ including limited provider understanding of FIM programs, ^41^ a desire for more engagement and information about FIM programs, ^48^ the learning curve to integrate new FIM programs into workflows, ^62,63^ and how to identify eligible patients. ^46,47^ Staff turnover and shortages were also challenges, which may result in lower FIM program screening and food distributions. ^47,49,55,60^

#### Individual Characteristics

Four sources described individual characteristic barriers to FIM programs (Table 1). ^41,44,45,67^ Few healthcare providers reported being comfortable providing patients with nutrition education due to a lack of training, apart from RDNs or providers that had formal nutrition seminars during medical school. ^41,45^ Varying levels of interest in FIM programming by providers contributed to challenges in identifying and recruiting participants. ^67^ Providers forgetting to bring the FV prescription pads to appointments was also a challenge. ^44^

## DISCUSSION

### Main Findings and Importance

Application of implementation science to FIM program integration in healthcare settings is important to understand gaps and opportunities among diverse contexts, which will help to improve the success of these efforts. ^23–26,29^ Findings from this narrative review provide a comprehensive account of FIM program implementation evidence to date using an implementation science framework, which resulted in an EPIS-informed^24,25^ implementation checklist for use by healthcare organizations/providers, implementing partners, and technical assistance personnel (Text Box 1). Several implementation strategies^22,23^ to facilitate FIM programs in the healthcare context were described, although this implementation science terminology was not explicitly used.

For example, in comparing narrative review results to the compilation of 73 implementation strategies for integrating innovations into healthcare settings, ^22^ we find at least 20 unique strategies reported to facilitate FIM program integration among multiple levels of influence. The most commonly used implementation strategies were “revise professional roles/create new clinical teams” regarding strategically delegating staff/team roles and procedures for FIM programs. ^18,39–43,46,47,49,51,53,54,57,59,60,64,66^ Training,^18,41,42,47,49,56,57,62,65^ developing tools or changing record systems for quality monitoring using EMR technology, ^39,42,48,49,54,60^ “facilitation” or interactive problem solving, ^38,47,49,57,59^ obtaining formal commitments, ^18,42,53,54^ reminding clinicians, ^43,44,46,57^ and identifying and preparing champions were also implementation strategies used more often. ^40,54,59,65^ However, reviewed sources were not specifically designed to evaluate the outcomes of these applied implementation strategies, which is a prime area for future research. FIM practitioners and researchers are encouraged to define, use, evaluate, and disseminate the outcomes of implementation strategies for FIM programs using standard implementation science terminology. This will allow for an improved understanding of which implementation strategies help to improve FIM program integration and how. ^22,23^

Additionally, only four reviewed sources^39,49,63,64^ utilized an implementation science lens. Another important finding from this work, therefore, is that the current literature is lacking a depth that would be useful to fully understand factors that influence the integration of FIM programs in healthcare settings. For example, EPIS *Individual Characteristics* findings largely centered around providers’ favorable perceptions of FIM programs. ^18,38,39,41,46,48,58,61,64^ While this information could be leveraged to encourage new healthcare settings to adopt FIM programs, we have limited understanding of providers’ social and demographic characteristics or other individual factors that may help or hinder FIM program integration in healthcare contexts. Overall, using standard implementation science terminology and theories, models, and frameworks to identify and describe barriers and facilitators and the types of supports needed for FIM programs can help to build the evidence on what works and under what conditions to inform scaling opportunities. ^23,27^ Given the noted importance of available or modified EMR technology systems to support screening, referrals, data accessibility, and closed-loop communication, technology-specific implementation science frameworks may be helpful to improve the technological capacity of healthcare organizations for FIM alongside EPIS or another suitable implementation science framework. ^28,37^ This call to action is aligned with the development of core competencies and continuous quality improvement goals in healthcare. ^26^

Formalized technical assistance centers are one promising avenue to support FIM researchers and practitioners in these efforts, regarding gathering, aggregating, and analyzing data and providing evidence-based guidance around FIM integration that builds on available assets and overcomes common barriers in healthcare settings. ^28^ For example, the NTAE^17^ and Feeding America Network^68^ use a technical assistance and evaluation approach to support over 100 FIM programs nationwide, and the National Institutes of Health aims to support similar strategies. ^69^ Organizational, state, and national policy support to embed technical assistance centers in the FIM movement is needed to aid FIM researchers and practitioners to standardize implementation science evidence and guide the use of the checklist (Text Box 1), for example, through the provision of support for technical assistance personnel around: human, financial, and infrastructure resources; knowledge to develop strategies and resolve issues; leadership; diverse partnerships; project management; engagement with communities; and workforce capacity and competency for program delivery. ^70^ Technical assistance providers could also lead the adaptation of available training manuals to help build opportunities for serving as FIM implementation facilitators, ^71^ as this approach seemed to be useful for FIM programs in the available literature.

### Limitations

Narrative review approaches are useful for providing an expert view of an emerging literature base and do not typically rely on systematic search criteria. ^30,31^ As such, supporting literature that meets our review scope may have been inadvertently overlooked; however, this limitation was mitigated by co-authors’ access to ongoing NTAE reviews using robust search methods and our knowledge of emerging literature on this topic. Scoping and systematic reviews will be useful in the future to synthesize a higher quality evidence base, grounded in implementation science theories, models, and frameworks, regarding the contextual factors that influence FIM program integration and the effectiveness of implementation strategies to support FIM program integration in healthcare settings.

Furthermore, characteristics of FIM programs, healthcare settings, and implementors varied in the identified literature and were not always consistently reported, making it difficult to discern if barriers and facilitators differed by these factors. It may be that certain healthcare settings serving patient populations with low income, such as Federally Qualified Healthcare Centers or those located in rural or tribal settings, for example, have added challenges or could benefit from different types of implementation strategies. Building a more nuanced implementation science evidence base will be important to help ensure equitable reach and expansion of FIM programs. ^28^ Furthermore, similar efforts to understand barriers and facilitators to FIM programs using an implementation science lens among implementing partner settings (e.g., food retailers, food banks/pantries) and focused on other EPIS domains will be useful for a holistic understanding of FIM program integration and scaling needs.

## CONCLUSIONS

FIM program integration in healthcare settings is challenging. However, several facilitators, including more than 20 implementation strategies, ^22^ have been helpful for supporting the integration of FIM programs in healthcare settings. The EPIS-informed checklist can be used as preliminary evidence in support of FIM program integration in healthcare settings. However, future research and practice approaches that ground efforts in the implementation of science terminology and theories, models, and frameworks are critical to build the evidence base around effective strategies to integrate FIM programs in healthcare settings across a wide range of contexts. Policy decisions that support resource and capacity building among FIM-implementing organizations are also warranted. As national conversations about the financial sustainability of FIM programs ensue, the capacity and best-practice workflow of the healthcare organizations involved should be considered. It would be remiss not to mention concerns about over-medicalization of access to healthy food, ^72^ given the lack of universal access to healthcare and overburdened US healthcare systems. FIM programs are meant to support acute or short-term healthy food access, whereas sustainable, systemic, and equitable access to healthy food must also be addressed outside of healthcare settings to be more holistic and sustainable.

## Supplementary Information

Below is the link to the electronic supplementary material.

Supplementary file1 (DOCX 72 KB)
