# Supplementary material for: Implementation of Food is Medicine Programs in Healthcare Settings: A Narrative Review
Source: J Gen Intern Med. 2024 Apr 25;39(14):2797–805. doi: 10.1007/s11606-024-08768-w (PMC11535093; doi:10.1007/s11606-024-08768-w)
Supplement: Supplementary file 1 — Supplementary file1 (DOCX 72 KB) [file 11606_2024_8768_MOESM1_ESM.docx]

| Supplemental Table 1. Characteristics of Sources^*^ Included in a Narrative Review about Barriers and Facilitators to Food is Medicine (FIM)^†^ Program Implementation in the Healthcare Context (n=31 sources). | | | | | | |
| --- | --- | --- | --- | --- | --- | --- |
| Author, Publication Year  Source Type | Objective | Study Design | Location Characteristics | Partnerships | Innovation | Clinic/Provider Characteristics |
| Aiyer et al., 2019^37^  Peer-reviewed article | To examine the feasibility, acceptability, cost, and preliminary impact of a collaborative pilot food prescription program in Harris County, TX. | Pre-post mixed-methods evaluation. | North Pasadena, TX–high rates of food insecurity and childhood and adult obesity. Also, a majority Hispanic population with lower levels of education and high percentage of families experiencing poverty. | Harris County BUILD Health Partnership including a local health department, food bank, and hospital. | Providers issued a “Food Rx” card for adults who screened for food insecurity, that included 30 pounds of fruits and vegetables (FVs) and 4 nonperishable products to use at a food pantry every 2 weeks for up to 6 months. | The program was implemented in two school-based clinics and 1 federally qualified health center (FQHC).  The type of clinic providers/staff were not described. |
| All in Alameda County, 2019^39^  Toolkit | A toolkit was created for primary care clinics and other healthcare settings that want to incorporate FIM initiatives into their practice and workflow. | Not applicable. | Features examples and resources specific to Alameda County, CA. | Recommends partnerships with community residents, local  growers, and community-based and food justice  organizations. | Various program types were mentioned: monetary vouchers (usually $5-$10), prescriptions for food, or home delivery of Community Supported Agriculture (CSA) boxes. Additional program components could include health fairs, cooking classes, referrals to local, state, or federal nutrition assistance, and food “farmacies” or farmers’ markets at clinics. | The toolkit was intended for clinics that serve a pediatric population. |
| Atcheson, 2018^60^  Thesis | To evaluate fidelity of Wholesome Wave Georgia’s (WWG) FV Prescription Program (FVRx) with a new program plan at Grady Health System and to review the existing literature regarding best markers for program sustainability. | Implementation process evaluation. | Metro-Atlanta, GA. | Grady Health System, WWG, Open Hand Atlanta, Common Market Produce,  and Fresh MARTA Markets. | Healthcare providers wrote produce prescriptions for patients with chronic diseases and registered dietitian nutritionists (RDNs) provided monthly group nutrition education and weekly Cooking Matters curriculum. | Those involved in program implementation at the clinic level included a lead RDN, Grady RDNs, support staff such as dietetic interns and diet technicians. |
| Auvinen et al., 2022^38^  Peer-reviewed article | To identify perceived facilitators and barriers for designing and implementing produce prescriptions within the healthcare system. | Qualitative. | Not applicable. | Not applicable. | Not applicable. | Two clinicians were included in broader in-depth interviews that also included Medicaid representatives (as one potential payer) and other partners relevant to produce prescription programming. |
| Balis et al., 2024^63^  Peer-reviewed article | To explore 1) perceptions of public health impacts of perinatal FIM programs from the perspectives of both program implementers and program supporters, and 2) implementation strategies used to enhance program adoption, implementation, and maintenance. | Qualitative. | Diverse geographic areas across the United States. | Not applicable. | Perinatal FIM programs. | Program implementers (organizations with active perinatal FIM programs) and program supporters (policymakers, funders, implementation partners), including those from healthcare organizations. |
| Budd Nugent et al., 2022^49^  Peer-reviewed article | To describe the implementation of two U.S. Department of Agriculture, Gus Schumacher Nutrition Incentive Program (GusNIP) funded produce prescription programs in two rural tribal communities. | Descriptive case study. | Yukon-Kuskokwim Delta, AK–remote tribal community with a high proportion of persons participating in Supplemental Nutrition Assistance Program (SNAP), high rates of chronic disease, and low food access/accessibility due to remote location and harsh climate. | Tribal healthcare, food wholesale, and grocery. | Eligible patients (with risk for or diagnosed with diabetes, and Medicaid insurance) received prescriptions (up to 24 for $45) for fresh, frozen, or canned FVs. Culturally appropriate recipes and cooking demos were also provided. | The Yukon-Kuskokwim Health Corporation, one of 12 tribal healthcare delivery systems in Alaska, that served 58 remote villages and over 23,000 individuals through a main Yukon-Kuskokwim Delta Regional Hospital in Bethel, AK, as well as five subregional clinics and 41 village clinics located throughout a 75,000-square mile area. |
|  |  |  | The Navajo Nation, spanning NM, AZ, and UT–a remote area with high rates of food insecurity, unemployment, and chronic disease, and few grocery stores. | Food retail, healthcare, tribal health programs, early childhood education, community-based organizations, and a technical assistance provider. | Eligible participants (diet-related risk, food insecurity, and pediatric or expecting persons with lower income) attended monthly health coaching sessions and received vouchers (up to $4/day per household) for traditional fresh, frozen, or dried FVs. | 15 healthcare facilities (no other details). |
| Coward et al., 2021^40^  Peer-reviewed article | To examine the attitude towards food prescription (FRx) interventions among clinicians and identify potential barriers to their use in clinical practice. | Qualitative. | North and Central MS (Oxford, Tupelo, Batesville, Jackson, Charleston) and New Orleans, LA – primarily populations with lower income and located in more rural areas. | Not applicable. | Not applicable. | The study sample included fifteen healthcare providers: three physicians, four RDNs, three nurses, and three nurse practitioners (two providers not described). Sample included those with mixed familiarity with FRx and represented providers that were predominately white, female, 26 to 38 years old, and with 3 to 27 years in current profession (a majority having >10 years of experience). |
| DePuccio et al., 2022^41^  Peer-reviewed article | To examine the barriers to and facilitators of implementation regarding a food referral program offered to primary care patients screening positive for food insecurity and with a qualifying chronic condition (i.e., diabetes, obesity, hypertension). | Qualitative. | OH–Urban area serving a high proportion of patients with lower socioeconomic status. | The Mid-Ohio Farmacy (MOF), a food referral program implemented collaboratively between a regional food bank and partnering healthcare providers. | Adult patients and family members received weekly fresh produce from one of sixteen participating food pantries. | Two family medicine clinics affiliated with a large academic medical center. Study participants included twenty healthcare providers in MOF-affiliated clinics (i.e., physicians, residents, nurse practitioners, and pharmacists) and academic medical center administrators. |
| Folta et al., 2023^64^  Peer-reviewed article | To understand factors related to adoption and implementation of produce prescription programs. | Qualitative comparative case study. | South, Northwest, Midwest, and Northeast regions. | National non-profit organization and healthcare. | Enrolled participants received monthly financial incentives to purchase produce at grocery stores. Dollar amount, program length, nutrition education components, and population served varied by program. | Interviews with 8 clinic staff from 5 primary care safety net clinics. |
| Friedman et al., 2014^58^  Peer-reviewed article | To explore the influence of health care provider communication and role modeling for patients diagnosed with diabetes within the context of a farmers’ market located at a FQHC. | Mixed methods | SC–a majority African American population with a high prevalence of residents experiencing poverty. | A community-based participatory research approach including a farmers market managed by a local community member. | All FQHC patients were eligible to receive a prescription redeemable for $1 per visit. Patients were also enrolled in a diabetes education program at and received a $5 voucher after attending diabetes self-management education classes (four total). | The FQHC site opened in 1969 and was described as one of the largest rural community health centers in the state. Nine of 13 participating FQHC providers and ancillary support staff completed a 19-item online survey (3 physicians, 4 nurse practitioners, 1 health educator, and 1 social worker). |
| Garfield et al., 2021^42^  Report | To identify key challenges that currently inhibit the growth of produce prescription programs and provide recommendations for policies at the federal, state, and institutional level that could support their expansion. | Qualitative. | Not applicable. | A noted need for collaboration across sectors—including health care, social services, retail, and others. | Not applicable. | Sixty-two interviews with collaborators from produce prescription programs, including health care providers (and non-healthcare collaborators). |
| Goddu et al., 2015  Peer-reviewed article | To describe the development of the Food Rx program as well as preliminary implementation lessons learned. | Descriptive case study. | The South Side of Chicago, IL–a predominantly working-class African American community with limited healthy food access and disproportionately high rates of diabetes compared to the rest of the city. | Academic researchers, physicians, community experts, Walgreens, health centers, and a farmers market. | The Food Rx program combined a prescription, a coupon, nutrition information, and a map of participating Walgreens and farmers market locations for redemption. Providers gave patients a visually appealing, low-literacy resource during clinic visits. | The six implementing clinics collectively cared for approximately 10,000 patients with diabetes, who were noted to struggle with diabetes management. Four of the six participating clinics were described as members of larger clinic networks. |
| Hager et al., 2022^43^  Report | To describe results from the Food is Medicine 2.0 (FIM 2.0) project, a fifteen-month initiative that supported food bank-health care partnerships. | Project evaluation. | Programs located in: Atlanta, GA; Louisville, KY; Richmond, VA; Cincinnati, OH; Indianapolis, IN; Houston, TX; Nashville, TN; Riverside & San Bernadino counties, CA; Northern Nevada; Northwest Indiana; Baton Rouge, LA; Cleveland, OH; St. Louis, MO; and Eastern Iowa. | 14 food bank-health care partnerships. | An effort aimed at  connecting people facing hunger to food distribution programs for healthy food. The program engaged hospital outpatient  clinics to support clinic staff in conducting universal food security screenings, offering referrals, and supporting the development of promising interventions and activities. | Healthcare partners of food bank grantees. |
| Johnson et al., 2023^44^  Peer-reviewed article | To explore pediatric clinicians' experiences after enrolling patients in a novel produce prescription program in an urban primary care clinic. | Qualitative. | Washington, DC. | Clinics, community members, local farmers. | Families who screened positive for food insecurity or described a food hardship were offered standard resources and the option of being enrolled in a free 8-week food delivery prescription program (4 home deliveries of fresh produce). | Thirteen pediatric primary-care clinicians referred families into the program. Eleven were interviewed, including eight physicians and three nurse practitioners. Half had been in pediatric practice for more than 15 years and three-quarters for three or fewer days per week. All clinicians were female; 5 were white, 4 were African American/Black, and 2 were Asian. |
| Joshi et al., 2019^45^  Peer-reviewed article | To present experiences and lessons learned and provide guidance for building linkages between health care settings and community organizations. | Implementation process evaluation. | Cuyahoga County, OH–neighborhoods with a majority African American population living in low income and low food access areas. | County-wide collaborative including academic, public health, clinic, and Extension partners. | Produce prescription program for hypertension (PRxHTN) screened patients with hypertension for food insecurity. Blood pressure checks, nutrition counseling, and four, $10 farmers market produce vouchers were provided at each of three visits. | Three safety net clinics with implementation teams that varied across sites: (1) pharmacist lead with pharmacy technician support; (2) nurse care coordinator lead with nurse care coordinators’ support; and (3) business manager lead with two medical assistants and a student nurse practitioner support. |
| Levi et al., 2023^65^  Toolkit | To describe approaches that help facilitate the healthcare system to provide nutritious food, or resources to access nutritious food, to high-risk patients. | Not applicable. | Not applicable. | Healthcare systems, public health, and community partners. | FIM programs. | This resource was intended to summarize ways that healthcare systems, public health practitioners, and public health allies can partner to address diet-related health disparities. |
| Marcinkevage et al., 2019^46^  Peer-reviewed article | To describe mixed-method process and outcome evaluation results after two years of implementing a FV prescription program. | Mixed methods process and outcome evaluation. | Washington state–a state reported to have food insecurity disparities across counties, with some locations documented to have food insecurity prevalence 1.5 times the national average. | Washington Department of Health in partnership with public and private health care systems, public health agencies, a community-based organization, and a supermarket chain. | In general, a FV prescription for $10 was provided and could be redeemed at one of 169 participating supermarkets (program engagement, prescriptions, and dosing varied among partners). | Providers (e.g., RDNs, nurses, social workers, health educators, community health workers, clinicians, and outreach workers) across 14 implementing sites, including: three FQHCs; two general hospitals; one pediatric primary care clinic; one outpatient medical clinic; one public hospital district; one tribal health department; four local health departments; and one community-based organization. |
| McWhorter et al., 2022^48^  Peer-reviewed article | To understand the barriers and facilitators for healthy eating among a diverse population with diabetes and low income and the gaps in knowledge and training needs for RDNs to address patient barriers when  implementing a food prescription and Culinary Medicine program in a healthcare setting. | Qualitative. | Houston, TX–food insecurity prevalence is noted as above the state and national averages. | The University of Texas Health Science Center at Houston School of Public Health, in partnership with Harris Health System and the Houston Food Bank. | Prescription for Healthy Living, a 2-year initiative, that included co-creation, implementation, and evaluation of a comprehensive food prescription and culinary medicine program for patients with food insecurity and type 2 diabetes. | The Harris Health system was noted as serving one of the most impoverished geographic areas in TX.  Three RDN focus groups included 17 of the 29 employed RDNs. |
| McWhorter et al., 2023^47^  Peer-reviewed article | To determine perceived benefits and implementation barriers of the Houston Food Bank (HFB) food prescription program among clinicians and other clinic staff members. | Qualitative | Houston, TX–food insecurity prevalence is noted as above the state and national averages. | Food bank and healthcare clinic partnerships | An HFB operated food prescription program, Food Rx (no additional details provided). | The program operated with seventeen health care clinics (i.e., FQHCs, charity clinics, health systems, a city health department, and an academic health center). A convenience sample of 252 health care staff members from a variety of roles across organizations responded to a survey. |
| Poulos et al., 2021^52^  Peer-reviewed article | To explore the challenges and facilitators to implementing partnerships between food banks and healthcare, specifically from the perspective of food banks. | Qualitative. | TX. | Food bank and healthcare partnerships. | FIM or produce prescription interventions, broadly. | Eight of 21 TX food banks were identified as having an active food bank/healthcare partnership in 2020. |
| Poulos et al., 2023^51^  Peer-reviewed article | To identify and describe food bank-healthcare partnerships, the impetus for development of partnerships, and challenges to sustainable partnerships. | Qualitative. | TX. | Food bank and healthcare partnerships. | FIM or produce prescription interventions, broadly. | 21 food bank representatives who partnered with healthcare. |
| Reinoso et al., 2022^66^  Peer-reviewed article | To describe lessons learned in food pantry partnership, fundings, logistics, and sustainability in a collaborative food access model integrated into healthcare. | Descriptive case study. | An Indianapolis, IN neighborhood that serves a large population of new immigrant families with a high prevalence of food insecurity. | Food bank, food pantry, and healthcare clinic partnerships. | Patients with food insecurity received a paper referral to present for registration at the on-site food pantry. Participants also had access to bi-lingual nutrition education programming. | The program operated out of an FQHC, which is part of the largest FQHC network in Indiana that delivers primary care and a broad range of other medical and social services. |
| Schlosser et al., 2019^53^  Peer-reviewed article | To report results of a qualitative process evaluation of PRxHTN that included a majority older African American adult population diagnosed with hypertension and experiencing food insecurity. | Qualitative. | Cuyahoga County, OH. | Health Improvement Partnership-Cuyahoga consortium including three safety net clinics and 20 farmers markets. | PRxHTN screened patients with hypertension for food insecurity. Blood pressure checks, nutrition counseling, and four, $10 farmers market produce vouchers were provided at each of three visits. | Five of 7 providers across three safety net clinics that implemented and delivered PRxHTN were interviewed (pharmacist, medical assistants, and patient care coordinators). |
| Share Our Strength and UCSF Center for Vulnerable Populations, 2022^50^  Toolkit | To offer a practical, user-friendly guide for planning and operating produce prescription programs in rural areas. | Not applicable. | Rural U.S. settings in general. | Informed by using surveys and interviews collected from rural produce prescription program operators and partners across the country. | Produce prescription programs, broadly. | Local, rural healthcare systems. |
| Short et al., 2023^67^  Peer-reviewed article | To assess the feasibility of a food-based diabetes self-management education (DSMES) and support intervention to persons with type 2 diabetes and food insecurity. | Mixed methods. | Southern AZ. | Food bank and health clinic partnership. | The intervention included a bi-monthly food package consisting of high-fiber/low-refined carbohydrate foods, recipes, DSMES materials, and 2 visits with an RDN. | 5 staff members from the food bank and FQHC completed interviews. |
| Smith et al., 2021^54^  Report | To describe results and key learnings from a FIM project, a two-year initiative that supported food bank and health care partnerships. | Project evaluation. | Programs located in: Atlanta, GA; Louisville, KY; Richmond, VA; Cincinnati, OH; Indianapolis, IN; Houston, TX; and Nashville, TN. | 7 food bank and health care partnerships. | Components of FIM programs varied by site, but broadly included screening patients for food insecurity and providing resources to reduce barriers to healthy food access. | Healthcare partners of food bank FIM grantees. |
| Stotz et al., 2022^18^  Peer-reviewed article | To investigate barriers, facilitators, and best practices for produce prescription program improvement among health care practitioners (HCPs) who participated in GusNIP between 2019-2020. | Qualitative. | GusNIP produce prescription projects from across the country, including those located in northeast, southeast, southwest, northwest, and AK/HI regions. | Not applicable. | GusNIP produce prescription programs, broadly. | Interviews included 16 HCPs identifying as women, majority white, and in practice for more than 10 years, including: case managers/care coordinators; health educator; medical doctor; nurse practitioner/physician assistant; nursing professionals; RDNs; social workers; and “other” providers. |
| Sundberg et al., 2020^55^  Peer-reviewed article | To describe a community-based participatory methodology used in the inception, design, and implementation of Navajo FVRx and provide key lessons learned. | Descriptive case study. | Navajo Nation–a sovereign nation that is geographically isolated with a limited number of food retailers, and noted to have the highest rates of food insecurity in the nation. | Local health facilities, local retailers (grocery stores, convenience stores, trading posts, and farmers markets), community-based organizations, national organizations, tribal health programs, and Navajo families. | Navajo FVRx enrolled families with a pregnant or postpartum mother diagnosed with diabetes or with a child between 3-6 years of age with overweight or obesity. The six-month program included nutrition education and vouchers based on family size (capped at $4/day for one month). | Implementation teams were based in clinical and community settings and included community health providers (e.g., public health nurses), clinicians (e.g., physicians, dietitians) and public health program employees (e.g., Head Start). |
| Trapl et al., 2017^61^  Peer-reviewed article | To examine feasibility of integrating a produce prescription (PRx) program into provider practices, ease of use of prescriptions and program materials, and the use of farmers markets by program participants. | Mixed methods. | Cuyahoga County, OH–neighborhoods with limited healthy food access, residents with lower incomes, and a high proportion of children living below the poverty level. | Partnership representing public health, Extension, and academia. | The 4-month PRx program issued participants monthly produce vouchers ($40) and providers recommended monthly nutrition counseling. | Interviews were conducted across four provider sites (FQHC; Special Supplemental Nutrition Program for Women, Infants, and Children (WIC) sites; community health center) and included four community health workers, one ‘supervisor,’ one RDN, three midwives, and one patient advocate. |
| Vericker et al., 2021^56^  Report | To summarize findings from a process evaluation examining Food Insecurity Nutrition Incentive (FINI) program models, implementation approaches, and experiences of grantees, participating retailers, and incentive issuance and redemption patterns. | Implementation process evaluation. | In general programs were located across 32 U.S. states in neighborhoods with high rates of low income, unemployment, and SNAP participation. | Not applicable. | Produce prescription programs varied across sites, although typically enrolled participants of SNAP in a healthcare setting and provided prescription vouchers for redemption at participating retailers. | Twelve FINI grantees who worked with healthcare partners. |
| Washtenaw County Health Department, 2016^57^  Toolkit | To assist local public health departments and their community partners (clinics, farmers markets, and funders) in using an evidence-based model to implement a FV prescription program. | Not applicable. | Washtenaw County, MI–noted as an area of the state with high rates of income inequality. | Recommended partnerships that connect philanthropies, the medical system, and the food sector. | Healthcare providers wrote “prescriptions” for FV that could be redeemed for $10 in farmers market tokens (up to 10 times or $100). | Toolkit described the role of clinics in produce prescription programs (no other details available). |

*There was substantial heterogeneity regarding the quality of source reporting in relation to data extraction categories; information was populated based on the information available in each source.

^†^Refers to FIM programs based in a U.S. healthcare context that screen and refer patients to healthy, unprepared foods.

| Supplemental Table 2. Facilitators^*^ to Food is Medicine (FIM)^†^ Program Implementation in the Healthcare Context Using the Exploration, Preparation, Implementation, and Sustainment (EPIS) Framework (n=28 sources). | |
| --- | --- |
| **Author, Publication Year** | **Leadership (n=14 sources)** |
| All in Alameda County, 2019 | - Identifying champions to help lead and coordinate, including a project champion (to manage the primary responsibilities of the project) and organizational change champion (to coordinate efforts and strategies on a higher systems level) were recommended. - Institutional leadership support was described as critical to intervention success and sustainability and could help the champion(s) get buy-in from other key stakeholders (e.g., staff, patients, community partners). |
| Budd Nugent et al., 2022 | - Sites in the Navajo Nation looking to implement produce prescription programs were described as needing to adhere to a provider manual, that included getting department or leadership approval to operate the program. |
| DePuccio et al., 2022 | - Support and leadership from program champions were described as essential to facilitating initial partnership and implementation, encouraging provider uptake, and promoting ongoing engagement among clinics. |
| Hager et al., 2022 | - The success of the program was noted as directly connected to the commitment, involvement, and enthusiasm of implementing healthcare partners. |
| Joshi et al., 2019 | - Finding commonalities and matching priorities to address concerns among medical leadership was a noted facilitator (e.g., medical leadership were more likely to support clinic participation in PrxHTN when understanding the program could benefit existing efforts to improve hypertension management and patient adherence). - Clinic implementation leaders were selected by clinic medical leadership who had knowledge of the diverse clinic roles. |
| Levi et al., 2023 | - Identify a clinical champion passionate about supporting food security to help create systems and processes. - When designing a FIM program, it is important to build engagement from key partners starting at the earliest stages (e.g., working with electronic medical record (EMR) systems is complex and requires buy-in from many partners). |
| Poulos et al., 2021 | - All interviewed food bank employees emphasized the need for leadership and staff at food banks and healthcare sites to have a strong desire to support and understand the interconnectedness of food insecurity with social determinants of health. - Program champions on both sides of a partnership were discussed as a facilitator. |
| Reinoso et al., 2022 | - The understanding and support of the physician-led senior leadership was essential to the program's success and sustainability. |
| Share Our Strength and UCSF Center for Vulnerable Populations, 2022 | - Meeting with clinic leadership and staff about the program and to establish buy-in at the beginning was described as key for success and sustainability. - Identifying and developing champions for a produce prescription program was often reported as key for long-term success and sustainability. |
| Smith et al., 2021 | - Working with an implementing physician “champion” was described as potentially increasing the health system’s focus on social determinants of health and related metrics. |
| Stotz et al., 2022 | - Hospital or health care administration were described as beneficial for overcoming data sharing, EMR abstraction, and IRB approval barriers for produce prescription programs through the provision of a letter of support for partner organizations and grant funders. |
| Sundberg et al., 2020 | - Finding ways to engage supervisors and leadership early on to increase support from them was described as a facilitator. |
| Vericker et al., 2021 | - Clinicians’ buy-in was noted as important for patient recruitment and retention. |
| Washtenaw County Health Department, 2016 | - Determining the best person to speak with (such as the clinic manager), requesting a meeting, outlining program requirements from clinic staff, and planning a persuasive and clear dialogue that aligns with leadership’s mission and strategic plan were described as facilitators for planning a produce prescription program. |
|  | **Organizational Characteristics (n=12 sources)** |
| Aiyer et al., 2019 | - The food prescription program reportedly strengthened ties with community programs that promoted healthy eating and/or healthy weight. - One provider noted the importance of building trust and communication between organizational partners. |
| All in Alameda County, 2019 | - Vision alignment was described as important at all levels of implementation leadership. |
| Balis et al., 2024 | - Perinatal FIM programming is compatible and aligns with the missions of existing organizations. |
| Budd Nugent et al., 2022 | - The Yukon-Kuskokwim Health Corporation (YKHC) infrastructure allowed for the use of satellite village clinics and telemedicine. - YKHC’s vision was noted as, “Through Native self-determination and culturally relevant health systems, we strive to be the healthiest people”, which was woven into day-to-day operations of the Diabetes Prevention and Control (DP&C) department and a foundational aspect of produce prescription activities. As one example, program-specific recipe cards were used to encourage participants to use subsistence foods with fruits and vegetables (FVs), traditional recipes, and cultural teachings. |
| Hager et al., 2022 | - Frequent communication between food bank and the health care partner(s) was described as important to program success. - The relationship between food bank and health partners was reported to have strengthened during the grant period because of the number of necessary interactions and progress updates. - Numerous health care partners were reported to have approached the food bank to replicate the model into their own network and bring food insecurity solutions to their communities. |
| McWhorter et al., 2023 | - Having a value-based care strategy to address food and health needs in populations vulnerable to food insecurity and recognizing the role of food prescription programs were described as facilitators. - Coordinated patient care within and between organizations was noted as useful for implementing an effective and standardized partnership-based food prescription program, including recognizing the value of establishing consistent communication channels between clinic and patients. |
| Poulos et al., 2021 | - Food bank employees highlighted the importance of mission compatibility and organizational readiness across both food banks and healthcare partners. |
| Poulos et al., 2023 | - Healthcare partners reached out to food banks to establish partnerships for programming. |
| Reinoso et al., 2022 | - Being a part of a larger health system brings benefits such as the utilization of the health center space, utilities, and additional infrastructure support. |
| Share Our Strength and UCSF Center for Vulnerable Populations, 2022 | - Executing necessary data sharing and partnership agreements (e.g., memorandum of understanding) was noted as an important facilitator. - It was recommended to explore partnerships with nontraditional rural health sites (e.g., school-based health center; local WIC agency) if no traditional healthcare system or clinic is located in a community where programming is desired. |
| Smith et al., 2021 | - A food bank and health care partner held monthly check-in calls to provide updates, problem solve, and plan for upcoming changes to facilitate programming. |
| Washtenaw County Health Department, 2016 | - Choosing a partner clinic that serves the produce prescription program priority population and in close proximity to a farmers market (redemption site) was noted as important. |
|  | **Quality and Fidelity Monitoring and Support (n=21 sources)** |
| Aiyer et al., 2019 | - Clinic staff received monthly conference calling to discuss implementation challenges and successes across sites, which was considered important and helpful. |
| Atcheson, 2018 | - It was recommended that program leaders track and formally discuss implementation successes and/or issues that arise to make appropriate decisions for future cohorts and to amend the program plan. This was describe as facilitating program sustainability and higher fidelity during expansion/replication. - It was recommended that a staff memo be sent out to all outpatient physicians about the program. - Intentional emphasis among providers regarding produce prescriptions as another “medication” prescription and encouraging patient redemption was noted as just as important as managing patients’ diagnosis and pharmaceutical medications. Patient fidelity to the program was noted as likely to decrease over time without this emphasis. - It was suggested to hire one registered dietitian nutritionist (RDN) specifically to coordinate programming to lead to higher fidelity, given one person would be responsible for shaping the flow and details of all program cohorts as opposed to multiple outpatient RDNs who are primarily responsible for patient care. |
| Auvinen et al., 2022 | - Clinicians (and others) described an EMR as an important implementation tool for produce prescriptions to prompt clinicians to screen patients for produce prescription eligibility, directly enroll or refer patients into a produce-prescription program, and track patient outcomes. - Clinicians (and others) described a need to equip clinics with resources and clear instructions for program implementation. |
| Balis et al., 2024 | - Using pilot programs as a trial run was a noted facilitator for influencing adoption. |
| Budd Nugent et al., 2022 | - A toolkit developed by two nonprofits required that implementing produce prescription sites had a team charter agreement that required follow-through with the program for a minimum of one six- or nine-month program cycle. - A unique strength of the produce prescription program was described as its integration with the Resource and Patient Management System of the Indian Health Service, a decentralized, integrated EMR for managing clinical and administrative information in tribal facilities, which shares a single EMR across Navajo Nation healthcare facilities and enabled widespread use of referral templates and data abstraction protocols. |
| DePuccio et al., 2022 | - Some medical assistants wrote notes in the EMR to remind physicians to discuss the program with the patients or to indicate that a particular patient expressed interest in learning more. - Program champions informed primary care staff about their referral rates and encouraged greater referral activity during patient visits. |
| Friedman et al., 2014 | - One provider suggested that monthly e-mails should be sent to providers to encourage them to distribute the farmers market prescriptions. |
| Goddu et al., 2015 | - The placement and design of the Food Rx shelves as well as regular check-ins and email updates aimed to serve as reminders of the program for providers. Every few weeks the shelves were restocked, providers were reminded to use the prescription, and feedback was collected from clinic staff. - All six clinics integrated Food Rx into their EMR systems to assist providers. |
| Hager et al., 2022 | - Recommended to prioritize working with one healthcare partner to pilot collecting health outcomes and these types of outcomes could help to strengthen partner support. - Becoming an agency partner enabled healthcare organizations to enter aggregate client demographic and health metric data through the food bank’s service insights system, which helped overcome the reality that large health systems require multi-layer approvals to provide any sort of data related to health. |
| Johnson et al., 2023 | - Provider suggestions included using electronic enrollment. |
| Joshi et al., 2019 | - Implementation fidelity at clinics was tracked using an electronic process tracking database (for number of clinic interactions and discussion topics) that was maintained by an academic partner, patient enrollment screen forms, a provider-prompted documentation sheet to encourage patient discussion and dietary counseling/goal setting, and a pre- and post- client survey including questions about provider interactions. - Formal and informal communication streams (e.g., monthly checklists to guide patient-provider interactions, constant communication between research staff member and implementation lead) during the implementation period helped to address challenges (e.g., IT troubleshooting, different priorities among clinicians and researchers). |
| Levi et al., 2023 | - Referrals are more likely to be successful if the healthcare center actively enrolls participants onsite or in the clinic, provides a warm handoff directly to resource representatives, and creates a program that is accessible and aligns with patient’s needs and preferences. - Integrate screening and referral processes into existing workflows and data systems so that it causes as little additional work for clinical providers as possible. - Obtaining EMR data may be easier for healthcare partners that are implementing FIM programs within the walls of their system due to privacy laws. |
| McWhorter et al., 2023 | - Quality improvement metrics for clinicians to understand their current performance in screening and responding to patient needs associated with social determinants of health was noted as helpful. |
| Poulos et al., 2021 | - Food bank employees suggested that healthcare partners should universally screen all patients to ensure equitable access to program resources. |
| Reinoso et al., 2022 | - Streamlining and decreasing the human resource burden of screening and resource referral through existing workflows in the EMR was essential. - Development of a dashboard for social determinants of health data collection was another approach to sustaining the workflow process and high screening rates. |
| Share Our Strength and UCSF Center for Vulnerable Populations, 2022 | - Establishing the ability to collect health data (participant surveys vs. EMR) and establishing mutually agreed upon outcomes of interest among all partners were noted as facilitators. |
| Smith et al., 2021 | - The health care partner assigned a staff member to manage data reporting, which led to successful data sharing. |
| Stotz et al., 2022 | - Best practices to facilitate health care providers referring patients to produce prescription programs included using validated screener questions in the standard medical intake form to ‘flag’ eligible participants to a full-time program staff person. Using diverse patient marketing techniques was also recommended (e.g., text message blasts, clinic marketing materials). - To overcome challenges with patient engagement (e.g., reminders for nutrition education, voucher issuance/redemption, follow-up visits), hiring a full-time staff member dedicated to managing all aspects of the produce prescription program (does not have to be a clinician) is recommended. |
| Sundberg et al., 2020 | - Using EMRs to identify and flag patients and families for the produce prescription program, while confirming eligibility during provider visits, was a noted facilitator. |
| Trapl et al., 2017 | - The research team provided monthly technical assistance (about 8 hours/month) to the providers delivering the program. - Providers thought that the program provided an interface to get feedback from participants’ progress on FV consumption and nutrition counseling during monthly follow-up visits. |
| Washtenaw County Health Department, 2016 | - Use of an online scheduling tool viewable to all clinic partner staff was noted as helpful for planning patient enrollment sessions and could ensure an adequate staff-to-patient ratio. - Health department staff sent clinic partners monthly emails with the number of patients referred to the produce prescription program, the number of patients enrolled, and the farmers market visit rate. Some clinics requested individual market visit rates and reached out to patients to encourage program use. - RACI (Responsible, Accountable, Consulted, and Informed) was a recommended quality improvement tool to ensure uniform program implementation across all clinic partners. Clinic partners were recommended to assign the personnel accountable for program actions and to have them sign an agreement. |
|  | **Organizational Staffing Processes (n=23 sources)** |
| Aiyer et al., 2019 | - A learned best practice was involving the entire clinic staff in providing prescriptions, partner collaboration, and having a coordinating team. |
| All in Alameda County, 2019 | - Identifying or hiring program champions to help lead and coordinate the project was considered beneficial for program success. Champions were noted as needing support and participation from a variety of staff members, including staff that will interface with patients, screen, refer, track referrals, evaluate the intervention, and more. - Allied health professionals (e.g., Certified Nursing Assistants or Medical Assistants) were noted as potentially more effective team members to conduct screening than the healthcare providers. - Having the staff accustomed to screening all patients was noted as helpful for normalizing the process of asking questions about food access in the clinic for both staff and patients. Asking questions about food insecurity during a medical appointment was also described as potentially stigmatizing, so training staff who will be screening families was noted as critical as well as creating a clinic culture that makes families feel more comfortable. |
| Atcheson, 2018 | - Defining program roles among staff members and holding members accountable to individual duties was described as vital to maintaining the core aims of the intervention. - Maintaining the appropriate number of staff during program growth was noted as important for assessment, data collection, produce distribution, or other program needs. - It was recommended that incoming outpatient resident physicians be educated about the program and about how to discuss the program with their patients. |
| Auvinen et al., 2022 | - One clinician enrolled patients into the produce prescription program during patients’ normal nutrition counseling visit (i.e., not using a separate visit), which was noted as improving productivity. |
| Budd Nugent et al., 2022 | - A toolkit developed by two nonprofits required that produce prescription sites had a team of dedicated healthcare providers, including women’s health, pediatric, and health promotion specialists, to provide referrals, produce prescriptions, and track clinical outcomes and commitment to initial and ongoing training. - Yukon-Kuskokwim Health Corporation (YKHC) used community health aides (CHAs) in each village, which was where the bulk of medical care was reported to be provided. CHAs, most of whom were residents, were referred to as the “eyes and ears” of the remote health system and already provided chronic disease prevention and health promotion, so program implementation could be integrated into the existing workflow. |
| Coward et al., 2021 | - One healthcare provider described that it would be helpful to have additional staff (e.g., diet tech, diet aid, diabetes assistant) to do more in-depth questioning before sending in the educator or the clinician. - Several mentioned the need for training across disciplines (nurse, RDN, nurse practitioners, physician), the hiring of specialized staff to assist with on-boarding (population health nurse), weekly or monthly educational seminars, and the need for a state or federal agency to take on the responsibility and cost of systemic training. Healthcare providers also mentioned specific topics where more education was needed, including (1) types and use of screening tools, (2) community education, (3) potential benefits of such an intervention and (4) intervention logistics. |
| DePuccio et al., 2022 | - Medical assistants played an important coordinating role by screening, enrolling, and educating patients. - Providers explaining to patients that the purpose of the program was to specifically address access to nutritious foods seemed to help minimize stigma and facilitate implementation. |
| Folta et al., 2023 | - Clinics that utilized non-physician or non-nursing staff to implement the program reported better workflows. |
| Goddu et al., 2015 | - At one clinic, medical assistants had access to the program, which included a food prescription, a coupon, and a map of where to redeem the coupon, and gave it to providers when they saw patients with diabetes who were good candidates for the program. |
| Hager et al., 2022 | - It was noted that providing financial assistance or another type of capital to healthcare partners to compensate for the partners’ investment of time and effort could be a facilitator. - Recruiting students from the local nursing college to help staff and run the program was also noted as a facilitator for programming. - Providing flexible programming to accommodate participant needs (e.g., health care partner staff walked through the survey with patients who did not have internet/computer access at home, providing nutrition education via phone calls from dietitians rather than through in-person education) was a noted facilitator. - Training new staff members to counteract staffing changes was noted as potentially helping to increase the number of screenings conducted and patients participating in programming. - Providers being able to accommodate non-English speaking participants’ native language was a noted facilitator. |
| Joshi et al., 2019 | - Clinic staff received didactic training, including patient counseling about healthy eating and farmers market use, federal and state food incentive programs at farmers markets, developing a process flow chart for program referral, and EMR program integration for appointment scheduling and blood pressure measurement follow-up. This included an additional one-hour meeting about clinic workflow for enrollment, scheduling, and follow-up. - Clinic support staff led implementation processes in alignment with standard clinic operating procedures. |
| Levi et al., 2023 | - Train clinical providers on rationale and workflows for addressing food insecurity in the clinical setting. |
| Marcinkevage et al., 2019 | - Having bilingual RDNs, nutrition educators, and other health care providers on staff to distribute FV prescriptions helped to improve program reach. |
| McWhorter et al., 2023 | - Improving within-clinic workflow integration, including building capacity for the program and involving more staff to reduce burden were facilitators. |
| Poulos et al., 2021 | - Food bank employees reiterated that healthcare partners must have the willingness to devote time and resources toward the partnership. - Food bank employees felt it was essential for healthcare partners to receive training on the significance of food insecurity and how to successfully identify food insecure patients using the Hunger Vital Signs. |
| Reinoso et al., 2022 | - Each clinical team member needs a clear process and role to effectively administer screening questions, review results, refer patients, and follow-up whether food access needs were met. Individual team members cannot meet the patients’ needs without a team approach. - Volunteers are an important resource required for managing and sustaining a food pantry in a health facility. |
| Schlosser et al., 2019 | - One clinic delivered program education in a group setting to overcome workload issues (which also allowed for a positive social space for patients). |
| Share Our Strength and UCSF Center for Vulnerable Populations, 2022 | - It was noted that programs should fit into already busy workflows, and it should be considered who administers the program (considering healthcare providers are often too busy with enrollment), what the referral system is, and if the program will be integrated into other services. For example: community health workers can play a vital role in rural produce prescription program implementation and evaluation; medical assistants, nutritionists or RDNs, medical or nursing students, dietetic interns, or health educators may be helpful for assisting with program administration. |
| Stotz et al., 2022 | - Healthcare providers considered the essential staff for implementing produce prescription programs included RDNs, nursing or health care techs/assistants, and social workers/case managers. - To overcome the general lack of time or staffing at produce prescription program-participating clinics, ongoing training for prescribing health care practitioners was recommended. |
| Sundberg et al., 2020 | - Partner facilitated inter-professional team development curriculum for program implementors, developed throughout implementation and modified based on team feedback, helped to clarify team roles and responsibilities and strategies for effective communication. - To ensure success and define implementation roles, it was recommended to: consider community health team readiness using checklists; define team member roles (e.g., who will do community outreach); encourage a team approach (e.g., trust, shared responsibility, open communication); consider a person in a public health setting (e.g., public health nurse) for team leader; align roles and responsibilities with existing workflows; assign specific roles to team members; consider team size (e.g., smaller for community based, larger for clinic based); include program duties in job descriptions; hire nutritionists/RDNs; and delineate who holds and writes/signs vouchers. |
| Trapl et al., 2017 | - Providers across all three program models (home visit model, individual clinic visit, group clinic visit) found the program to easily fit within their practice. |
| Vericker et al., 2021 | - Partners educated healthcare providers about the financial incentive program using various methods (e.g., info sheet, staff meeting discussions) and presented information in person to clinic staff regarding a lack of community access to healthy foods and impacts on patients’ health. |
| Washtenaw County Health Department, 2016 | - Community health workers were noted as important for carrying out produce prescription programs and do not necessarily need to be hired by the clinic. - A unified presence of a clinic partner with a community member (i.e., community health worker) in group enrollment sessions was considered beneficial to programming. - Identifying personnel within the clinic to fulfill certain program tasks was noted as important. It was also noted that program partners, such as clinic personnel, spent less than one hour/week on the program. - In-person training was recommended, including a review of the program goals/benefits, referral process, and enrollment process, for staff members. - It was noted that all clinic members, especially those interacting with patients regularly, should know about the program and be able to answer basic questions, or at a minimum, be aware of the program and know who to refer a patient to for assistance. |
|  | **Individual Characteristics (n=13 sources)** |
| Aiyer et al., 2019 | - Clinic providers reported being more aware of food insecurity in the community they serve because of the food prescription program. - Clinic providers reported high effectiveness and high satisfaction of the food prescription program. |
| All in Alameda County, 2019 | - Many new initiatives were described to start with a great idea from a passionate individual within an institution or organization or a “champion”. Champions were described to generate internal and external enthusiasm for programming and were noted as needing strong communication skills. |
| Auvinen et al., 2022 | - One clinician described the popularity of their produce prescription program among staff who were carrying out direct patient interaction (e.g., care managers, community health workers, and dietitians). |
| Coward et al., 2021 | - All but three providers said they would feel comfortable prescribing more FVs. |
| Folta et al., 2023 | - Personnel at the clinic were motivated to adopt the program given potential benefits to the patients. |
| Friedman et al., 2014 | - Most providers thought patients responded to the prescription program “positively” or “very positively” and that it was “easy” or “very easy” to administer the program, especially when they had access to prefilled prescription pads. - Eight of nine providers thought it was important or very important that the farmers market remain at the clinic. |
| Goddu et al., 2015 | - Providers reported very positive experiences, were almost uniformly enthusiastic about the program, and were grateful for a resource to effectively address barriers to healthy eating. |
| Johnson et al., 2023 | - Clinicians described the program as having a direct positive impact on their patients with food insecurity, whereas they expressed dissatisfaction with the other resources available for families who screen positive for food insecurity. - Providers experienced enhanced job satisfaction. - The most discussed clinician-perceived program benefit was described as improved provider self-efficacy in addressing food insecurity. - Many clinicians expressed an increased motivation to ensure that food insecurity screening was completed during the visit because they knew they had this resource to offer. |
| Joshi et al., 2019 | - Clinician’s enthusiasm and engagement helped with program sustainability and cultural appropriateness. |
| Schlosser et al., 2019 | - Some providers found the programming to be energizing/fun to deliver. - Providers believed the program helped them to communicate that they cared about patient wellbeing. |
| Smith et al., 2021 | - Health care partner staff had the perception that several patients who frequently used Emergency Department care improved their chronic disease management after project participation. |
| Stotz et al., 2022 | - Most health care practitioners indicated a positive experience offering a produce prescription program and said they would participate again. Most were satisfied with the program and believed it positively impacted patient care and appreciated the ability to address social determinants of health. - Most health care practitioners agreed or strongly agreed that produce prescription programs improved how or if providers talk with patients about healthy eating, that these programs were beneficial for patients, and should be used at other clinics. |
| Trapl et al., 2017 | - Providers reported more ability, interest in, and excitement about talking about FVs and believed the program helped to improve provider-patient relationships. - All providers were willing to continue the program in subsequent years if offered. - Providers believed the program had relative advantage compared to other food assistance program options. - Providers felt better equipped to advise program participants to eat more FVs, as the program provided resources that allowed participants to prioritize healthy eating. |

*There was substantial heterogeneity regarding the quality of source reporting in relation to EPIS *Inner Context* categories; information was populated based on the information available in each source.

^†^Refers to FIM programs based in a U.S. healthcare context that screen and refer patients to healthy, unprepared foods.

| Supplemental Table 2. Barriers^*^ to Food is Medicine (FIM)^†^ Program Implementation in the Healthcare Context Using the Exploration, Preparation, Implementation, and Sustainment (EPIS) Framework (n=26 sources). | |
| --- | --- |
| **Author, Publication Year** | **Leadership (n=3 sources)** |
| Auvinen et al., 2022 | - Clinicians described the need for leadership, staff buy-in, and enthusiasm for produce-prescription programs (which could be a barrier) to be successful. |
| Smith et al., 2021 | - It took longer to obtain buy-in from individual departments than from general hospital leadership. |
| Sundberg et al., 2020 | - Limited support from supervisors and leadership was described as a barrier. |
|  | **Organizational Characteristics (n=7 sources)** |
| All in Alameda County, 2019 | - FIM interventions were described as time consuming to plan for and resource intensive. Appropriate infrastructure was noted as required, regarding the location of program activities, adequate space and equipment for cooking classes, advertising strategies, and alignment with existing group care visits. |
| Garfield et al., 2021 | - It was recommended that program partners (e.g., health care partners) should acknowledge, address, and overcome racism and other biases inherent to their systems and institutions (e.g., via institutional policies and practices, advocacy priorities, and training) to ensure equitable access to produce prescription services. |
| McWhorter et al., 2022 | - Some registered dietitian nutritionists (RDNs) described issues and concerns with allocating enough resources needed to build a food pantry for the program, including costs, maintenance, and staffing to ensure success. |
| Poulos et al., 2021 | - Noted need for clear communication and discussion about food expectations when entering a partnership between charitable food partners and healthcare providers (e.g., food variability often creates frustration because clinics can have expectations that are unachievable by food banks). - Few healthcare partners were described to have adequate refrigeration to safely store food, which might limit the variety of fruits and vegetables (FVs)/foods in program food boxes that are distributed at clinic sites. - It was noted that clinics serving a small patient population could pose a barrier for program implementation led by food banks (e.g., may not be a priority for food bank partners). |
| Poulos et al., 2023 | - Most of the clinics and hospitals were described as not designed to support safe food storage and therefore struggled with fresh food distribution. - Administrative challenges included the length and complexity of the approval process required at some facilities and the perceived administrative burden from the health care provider, which hindered partnership and programming opportunities. |
| Reinoso et al., 2022 | - The on-site food pantry at the clinic experienced space constraints for food storage and pantry shopping as the program increased in popularity and size. |
| Smith et al., 2021 | - Working with smaller clinic pantries required significant planning and engagement among food bank operations teams to shift practices (e.g., smaller orders, more frequent deliveries). - Clinic partners had specific inventory requirements (i.e., greater emphasis on FVs, whole grains, lean proteins, and limited processed foods or items deemed less nutritious) and food banks needed to work to accommodate. - A clinic was building a Food Pharmacy and experienced construction delays that hindered implementation. |
|  | **Quality and Fidelity Monitoring and Support (n=17 sources)** |
| All in Alameda County, 2019 | - A lack of terminology specific to FIM and food insecurity in the EMR, as well as a need to improve processes of documenting, coding and billing these interventions in the EMR was described as critical to providing comprehensive care, obtaining population data for clinical resource planning, expanding reimbursement, advancing research and quality improvements, and sharing food insecurity assessments and interventions with outside entities. |
| Auvinen et al., 2022 | - Logging into a separate platform to view benefit redemption information was described as disruptive to clinical workflow and time consuming, whereas tracking the produce prescription through the EMR was considered a useful solution (e.g., ideally an ICD-10 code would be used within an EMR to send an e-prescription to the pharmacy). - Clinicians described integration of produce prescriptions into EMRs and/or retail point-of-sale systems as important, but difficult to implement due to time-intensity. - Embedding produce prescription metrics into an EMR was described to require re-programming, which was noted as difficult and costly. |
| Balis et al., 2024 | - Connecting with healthcare providers to access participant biometric data was a noted barrier. |
| Coward et al., 2021 | - Lack of standardized screening tools was a noted barrier. For example, some providers used established screening tools or objective health measures, some adapted or changed their screening tool or protocol, and others did not use any screening tool. |
| DePuccio et al., 2022 | - Limited feedback and information flow between food pantries and providers made it difficult for physicians to assess the impact and success of the program among patients. - A low priority for food insecurity and referrals in relation to other clinic priorities during client visits was described to lead to low patient understanding of the program even if referred. - Program communication gaps had a dampening effect on providers’ enthusiasm to enroll patients into the program as the initial excitement diminished about the program. |
| Garfield et al., 2021 | - Implementation costs related to evaluation, technology, and administrative oversight was a barrier. - Data sharing was described as a barrier, regarding navigating patient privacy laws and participant concerns over data sharing with government entities. |
| Hager et al., 2022 | - Several challenges to pulling timely data and sharing data were described: changes to software and subsequent technical difficulties; clinic contacts needing to request for Information Technology (IT) to pull reports; having EMR/EPIC access and gathering data; and data being collected and analyzed by hand in spreadsheets. - There was no EPIC reminder for nurses to schedule consultations for dietary and social work staff after screening, which was a barrier to programming. - There were issues with fully tracking the number of screened patients for food insecurity during visits due to a high volume of patients. |
| Johnson et al., 2023 | - More than half of clinicians desired closed-loop communication regarding referred families’ program enrollment status to feel connected and ensure their referrals were successfully transmitted. |
| Joshi et al., 2019 | - A single approach for electronic integration of the program was described as not possible due to differences in EMR systems between clinics. |
| Levi et al., 2023 | - A lack of interoperability between data systems and concerns over HIPAA may limit communication between partners and create barriers to workflow integration. - Many healthcare systems lack a formal list of all available community food resources, do not have a standard process for tracking and closing referral loops, and lack coordination with external community-based organizations. - Some electronic health systems do not support easy tracking of food insecurity screening results. - No existing quality indicators/quality improvement metrics was noted as a common implementation challenge. - Technology needed to efficiently administer the program and track data is costly. |
| Marcinkevage at al., 2019 | - Implementers reported difficulty tracking paper-based prescriptions. - Many prescribers distributed prescriptions outside of the clinic setting (e.g., at community nutrition classes), which had to be recorded on paper tracking sheets because they did not have access to an EMR. - Introducing an electronic tracking system was described as cost-prohibitive in sites with an EMR, due to involvement of outside vendors or IT staff. |
| McWhorter et al., 2022 | - RDNs and patients described concerns over a lack of communication about food and dietary information and the need for a clear administration-sponsored communication strategy to overcome issues such as reducing “mixed dietary messages” between providers and patients and clarifying implementation of programming for eligible patients. |
| Poulos et al., 2021 | - Food bank employees and charitable food system experts noted that even with data agreements, healthcare partners often did not collect complete data or did not provide timely data reports back to the food bank as outlined in agreements. - All interviewees mentioned that some clinics may not want to or have capacity to share individual-level data because of patient privacy concerns. - Interviewees discussed that healthcare partners need to have a similar understanding of protected patient data (e.g., some healthcare systems view sharing food insecurity screening results with the food banks as a violation of HIPAA, yet other partners willingly share this information). |
| Poulos et al., 2023 | - A challenge was the lack of formal processes for referrals between healthcare providers and the food bank. |
| Smith et al., 2021 | - Data collection and sharing between food bank and health care partners was reported as a large barrier for several reasons: health care partners were concerned about logistical, operational, and legal challenges for collecting and sharing data with food bank partners; there were technical challenges with building infrastructure and reports in EMR systems to select and share metrics of interest and track patient data efficiently and consistently; healthcare sites did not always assign staff for data management and reporting or establish rigorous, consistent standard operating procedures; and data extraction was a capacity/staffing challenge. - A paper referral and tracking system was intensive and difficult to manage. The clinic partner stated a preference to build streamlined, digital data management systems in the future. - Food banks worked with health care partners to refer patients to food pantries and distributions outside of the health care setting, although completing referrals and connecting patients to additional services was challenging to implement and track. |
| Stotz et al., 2022 | - Providers experienced challenges collecting and sharing patient outcomes data. |
| Vericker et al., 2021 | - Providers experienced challenges keeping participants engaged in the produce prescription program over time, due to nutrition classes and health check-ins that were required to receive the FV incentive. |
|  | **Organizational Staffing Processes (n=20 sources)** |
| All in Alameda County, 2019 | - Staff capacity was described as needing to be considered. - Continued support was reported as needed among staff regarding a systematized process to act whenever a family is identified as food insecure. |
| Auvinen et al., 2022 | - Clinicians described the importance of staff and resource capacity to ensure the program was implemented well. - One payer noted that staffing capacity was a primary challenge for produce-prescription enrollment, implementation, and follow-up. Community health workers were noted as potential assistants for patient enrollment but were also noted to have time constraints. - High cost of implementation due to increased staff time. |
| Balis et al., 2024 | - Healthcare providers were overburdened by other responsibilities and enrolling patients did not always fit into their workflow. - Programs that changed the type of service provided to patients (e.g., from medically tailored meals to produce bags back to meals) were logistically challenging regarding the client care team workflow. |
| Coward et al., 2021 | - There was concern about a lack of understanding among healthcare providers regarding what a food prescription intervention was, how it was used, and the logistics for administering. |
| DePuccio et al., 2022 | - Due to time constraints during patient visits, physicians needed to rely on other clinical staff to conduct food insecurity screenings and engage with those who screened positive. - Physicians described having a hard time addressing patients’ clinical needs and co-occurring food insecurity within the allotted visit time, making it difficult for physicians to integrate program enrollment into their clinical workflow. |
| Folta et al., 2023 | - Some clinic staff reported the program as time- and resource-intensive, which resulted in increased workloads and altered workflows. |
| Friedman et al., 2014 | - Limited time for providers to discuss the farmers’ market with patients was described as a barrier. |
| Goddu et al., 2015 | - Some providers did not remember that the program was targeted for patients with diabetes who lived on the south side of Chicago and faced food security challenges and sometimes encouraged patients who were inappropriate for the program to redeem a prescription. - Staff, such as RDNs, were enthusiastic about the program but unable to prescribe a food prescription without a physician’s signature. |
| Hager et al., 2022 | - Health care staff were described as extremely busy with patient care and screenings, so adding an additional layer of food box distribution was noted as a possible distraction. - Staff changes, including staff shortages and the hiring of temp nurses and social workers, resulted in few screenings and lower food distributions. - At times, health care providers had difficulty determining if patients met inclusion criteria for the program. |
| Johnson et al., 2023 | - Although the program was designed to reduce clinician administrative burden, clinicians repeatedly described a desire to be more engaged and informed about program details. |
| Joshi et al., 2019 | - Clinic staff time to implement the program was a major challenge, including physician time given busy schedules. - Staff turnaround was a commonly reported challenge during the short duration of the program. |
| Levi et al., 2023 | - A lack of staff capacity or time was a reported as a common implementation challenge, including the burden on providers of screening and referring for food insecurity, particularly when there are inadequate systems and workflows in place. |
| Marcinkevage et al., 2019 | - Prescriptions needed a hand-written expiration date and to be hand-counted and was described as increasing prescribers’ workload and introducing the potential for human error. |
| McWhorter et al., 2022 | - All RDNs anticipated challenges in allocating or scheduling their time to offer classes and develop culturally tailored and practical resources, including cooking tips and recipes. - Some RDNs described issues and concerns with allocating enough resources needed to build a food pantry for the program, including staffing. |
| Poulos et al., 2023 | - Staffing and frequent staff turnover within healthcare organizations was a noted challenge. |
| Schlosser et al., 2019 | - Providers experienced workload challenges in completing PRxHTN activities and there was limited time for one-on-one provider-patient interactions. |
| Smith et al., 2021 | - Medical assistants reported food insecurity screening was time consuming and may not have occurred with all patients. - Clinic staff were noted as needing to be responsible for referring patients to a pantry network because food bank program navigators were not initially approved by Harris Health to operate on-site. |
| Stotz et al., 2022 | - Some health care providers reported a primary challenge of limited time and training for produce prescription programs. |
| Sundberg et al., 2020 | - Barriers included unclear roles and responsibilities among team members, team turnover, strained decision-making, and the size of the team in conflict with the setting or workflow. |
| Vericker et al., 2021 | - Nutrition incentives were a relatively new concept for healthcare providers and therefore the providers had to learn how they worked, how to incorporate the incentive programs into their work with patients, and how to "dose" a patient with a prescription for incentives. |
|  | **Individual Characteristics (n=4 sources)** |
| Coward et al., 2021 | - Fewer than half of the healthcare providers felt comfortable with their role in nutrition education and advocacy with patients. Notably of the others who were comfortable, most had some training in nutrition (e.g., RDNs or nutrition seminars in medical school). |
| Friedman et al., 2014 | - Providers forgot to bring the produce prescription pads with them during appointments. |
| Garfield et al., 2021 | - Health care providers often received little formal nutrition education, which posed a challenge to integrating produce prescription programs into health care. |
| Short et al., 2023 | - Medical providers had varying levels of interest in the program, which made identifying and recruiting participants a challenge. However, one clinic had a provider who was championing the program and had enrolled many participants. |

*There was substantial heterogeneity regarding the quality of source reporting in relation to EPIS *Inner Context* categories; information was populated based on the information available in each source.

^†^Refers to FIM programs based in a U.S. healthcare context that screen and refer patients to healthy, unprepared foods.
